# Lost Work Due to Burn-Related Disability in a US Working Population

**DOI:** 10.3390/ebj5040041

**Published:** 2024-12-19

**Authors:** Jacob M. Dougherty, Hannan A. Maqsood, Christopher J. Rittle, Eva S. Blake, Zhaohui Fan, Bryant W. Oliphant, Mark R. Hemmila, Naveen F. Sangji

**Affiliations:** 1 Department of Surgery, University of Michigan, 2101 Taubman Center, 1500 E. Medical Center Drive, Ann Arbor, MI 48109, USA; hl6843@wayne.edu (J.M.D.); cjrittle@med.umich.edu (C.J.R.); bryantol@med.umich.edu (B.W.O.); mhemmila@med.umich.edu (M.R.H.); 2 Center for Healthcare Outcomes and Policy, University of Michigan, 2800 Plymouth Road, North Campus Research Complex, Bldg.16, Ann Arbor, MI 48109, USA; hannanahmadmaqsood@gmail.com (H.A.M.); fanluck@med.umich.edu (Z.F.); 3 School of Medicine, Wayne State University, 540 E. Canfield Avenue, Detroit, MI 48201, USA; 4 University of Michigan Medical School, 1301 Catherine Street, Ann Arbor, MI 48109, USA; bleva@med.umich.edu

**Keywords:** burn injury, lost workdays (LWDs), short-term disability, long-term disability (STD), economic impact

## Abstract

Background: Burn injuries can require hospitalization, operations, and long-term reconstruction. Burn-injured patients can experience short- or long-term disability. We investigated lost workdays (LWDs), short-term disability (STD), and long-term disability (LTD) in the 12-month period following a burn injury. Methods: An observational cohort study was conducted using a commercial claims database, IBM^®^ MarketScan^®^. Patients aged ≤65 years with an ICD-10 burn diagnosis from 2018 to 2019 were included. The International Classification of Diseases, 10th Revision (ICD-10), procedure and Current Procedural Terminology (CPT) codes were used to identify patients undergoing burn-related operations. Patients were mapped to data tables for LWDs, STD, and LTD for the 12 months pre- and post-injury. Paired t-tests were employed to compare the pre- and post-injury outcomes. Results: We identified 1745 patients with burn diagnoses. Of those, 263, 1449, and 1448 patients had data available for LWDs, STD, and LTD, respectively. STD and LTD were reported by 8.1% and 0.0% of patients in the 12-month period pre-injury, respectively, and 20.3% and 1.0% of patients in the 12-month period post-injury, respectively. Average days of STD increased from 3.70 to 9.34 days following injury. Conclusions: Burn injuries are associated with increased STD and LTD utilization. Quantifying the impact of burn injuries on patients’ work will help us understand the economic implications of burns, which is a key area in burn research.

## 1. Introduction

Injury is a significant source of economic hardship in the United States (US). The economic cost of injury totaled $4.2 trillion USD in 2019, estimated by assigning the costs for medical care, work loss, value of statistical life (i.e., an economic evaluation used to determine the financial benefits of mortality risk reduction,) and quality of life losses [1]. Over half of that cost was attributed to working-age adults, and over $69 billion was attributed to work lost [1]. When specifically looking at occupational injury, an estimated 1.8 million workers who experienced a work-related injury required emergency department (ED) treatment in 2020 [2]. The economic burden associated with lost income can also be compounded by the costs of the injury itself. The medical costs of a non-fatal injury that requires an ED visit have been shown to average $6620 in the 12-month period following the injury, with a range from $1698 to $80,172, depending on the type of injury [3]. Along with the economic considerations of lost wages and the costs of care, working-age individuals may struggle to maintain employment and independence in their lives post-injury [4]. For those injured at work, workers’ compensation funds may be available to provide funds for medical care and to cover lost wages [5]. In 2022, the estimated medical costs for non-fatal burn injuries resulting in hospitalizations exceeded USD $4 billion, with USD $367 million in work lost costs. The estimated medical costs for burn injuries treated in the emergency department and resulting in discharge during the same period exceeded USD $1.5 billion, with greater than USD $450 million in work lost [6]. However, the direct impact on patients, as well as employers in terms of work lost, has not been previously quantified.

The ability of burn survivors to return to work is often hindered by various factors [7,8]. Physical limitations that result from burn injuries, such as fatigue, chronic pain, and reduced range of motion can decrease job performance, especially in roles that are physically challenging. Psychological difficulties, including depression, anxiety, and diminished self-confidence, can impact the reintegration of the individual into the workplace [9]. Burn survivors may also experience workplace discrimination, while a lack of disability accommodations can hinder their return to work. Tailoring return-to-work recovery plans to individuals’ specific needs, involving psychologists, vocational counselors, and occupational therapists, has been shown to improve employment outcomes and facilitate a smooth transition back into the workforce [7].

The size and location of the burn have also been shown to be critical factors in determining a burn survivor’s capacity to return to work [10]. Patients with burns affecting the face or hands face greater challenges in returning to work due to the psychological impact of having a highly visible injury and/or disability due to hand burns. Psychological resilience has been found to be a vital factor in the successful return to work [10]. Research suggests that patients with high levels of motivation and self-efficacy are more likely to effectively reintegrate into the workforce compared to those with low psychological resilience. Along with psychological resilience, social support networks have been found to influence the successful transition to the workforce. These networks include friends, family, and local support groups, and provide practical guidance and emotional encouragement. These findings emphasize the necessity of rehabilitation techniques that address psychological well-being and physical health to optimize return-to-work outcomes [10].

An estimated 486,000 patients per year require ED care for a burn injury, and approximately 40,000 patients are hospitalized as a result of a burn injury [11]. When hospitalized for a major burn injury, patients may need wound care, physical and/or occupational therapy, and rehabilitation, and functional recovery can take months or years following initial injury [12,13,14,15]. Working-age individuals may require time off from work to recover from an injury or illness, or to help care for a spouse or dependent suffering from an injury and illness. When offered by employers, options to recoup some lost wages during this time off include paid vacation days, sick days, and short-term or long-term disability. Short-term disability is limited in duration to a maximum of one year in most cases, while long-term disability is paid on a monthly basis until the employee reaches the Social Security normal retirement age (SSNRA) or 65 years old [16,17].

We aimed to quantify the economic impact of lost work in a US working-age population following burn injury. Our evaluation included the lost workdays (LWDs) and the short-term disability (STD) and long-term disability (LTD) leave taken by employees in the 12-month period preceding and the 12-month period following their burn injury. We then report the rates of LWDs, STD, and LTD among workers with burn injuries. It can be assumed that burn injury, specifically the need for skin grafting following a burn injury, would lead to lost workdays and the use of short and/or long-term disability. However, this has not previously been quantified for those suffering from burn injury, which is a critical knowledge gap for occupational health research. In contrast, non-burn traumatic injury has been well studied, and there is a clearer understanding of the types of traumatic injury and the associated impact on a working-age population and their employers [18,19]. We hypothesized that burn injury would lead to increased lost workdays and utilization of short-term disability and long-term disability in a working-age population compared to a working-age cohort that experienced all the other potential causes of lost work, except burn injury.

## 2. Materials and Method

### 2.1. Data Source

Our observational cohort study used a commercial claims database from IBM^®^ MarketScan^®^. MarketScan^®^ collates healthcare data for privately insured Americans and is one of the largest collections of privately and publicly insured, de-identified patient databases in the United States [20]. This study utilized the IBM^®^ MarketScan^®^ Commercial Claims Database and the Health and Productivity Management (HPM) Database, which combines data on workplace absences, STD, LTD, and workers’ compensation claims [20]. The database pools information from large employers and does not uniformly represent all areas of the country.

### 2.2. Study Cohort

Patients aged ≤65 years with an ICD10 diagnosis code of burn injury between January 2018 and December 2019 were identified in the MarketScan^®^ Commercial Claims and Encounters Database. To ensure the inclusion of only those patients who had a new diagnosis of burn during the study period, any patients with a burn injury diagnosis or skin graft procedure in the preceding year were excluded. These patients were then mapped to data tables for lost workdays (LWDs), short-term disability (STD), and long-term disability (LTD) in the HPM database for the 12-month period before and after the burn diagnosis. The patients were also mapped to the workers’ compensation data tables. A subset cohort of patients who underwent operations related to their burn injury was identified using ICD10 procedure codes and Current Procedural Terminology (CPT) codes for burn-related operations, such as excisional debridement and skin grafting.

### 2.3. Variables

The patient demographics were captured, including age at the index visit for burn injury, sex, the region of residence, insurance plan type (low, average, and high cost-sharing), employment status at the time of injury, industry classification, discharge disposition for those who were admitted for their injury, and year of the index visit. Low cost-sharing plans typically cover a majority of the expenses, while high cost-sharing plans often require patients to pay more out-of-pocket. The presence of patient comorbid conditions was also captured utilizing diagnosis codes provided in the patient record.

### 2.4. Outcomes

Lost workdays and the use of short-term disability and long-term disability in the 12-month period following burn injury were compared to the baseline level of lost workdays and the use of short-term disability and long-term disability. The baseline was defined as the 12-month period preceding the diagnosis of a burn injury. A subset analysis was conducted for patients with a diagnosis of burn injury who required an operation for their injury, such as a skin grafting procedure.

### 2.5. Analysis

A paired t-test was used to compare the LWDs, STD, and LTD in the 12-month period prior to the burn diagnosis and the 12-month period after the injury. We followed the Strengthening the Reporting of Observational studies in Epidemiology (STROBE) guidelines in this observational cohort study.

### 2.6. Ethics Review and Approval

This study is classified as exempt by the University of Michigan Medical School Institutional Review Board, given the secondary use of data.

## 3. Results

We identified 1745 patients with a burn diagnosis during the study period who had data captured in the HPM database. Patient demographics are included below; patients were most commonly between the ages of 25 and 44, male, employed full-time, and discharged to home following their injury (Table 1).

Of all the burn injury patients, 263, 1449, and 1448 patients had data available for lost workdays (LWDs), short-term disability (STD), and long-term disability (LTD), respectively (Table 2). Overall, LWDs were reported by 50.6% vs. 53.6% of individuals in the 12 months pre- and post-burn injury, with average LWDs of 5.15 vs. 7.99 days (*p* < 0.05). STD was reported by 8.1% vs. 20.3% (*p* < 0.0001) of patients in the 12 months pre- and post-burn injury, with the average days of STD being 3.70 vs. 9.34 days (*p* < 0.0001). LTD was reported by 0.0% vs. 1.0% (*p* < 0.0001) of patients in the 12 months pre- and post-burn injury, with the average days of LTD being 0.0 vs. 2.01 days (*p* < 0.01) (Table 2).

For patients who underwent a skin grafting procedure, 11 patients had data available for LWDs and 60 patients had data available for STD and LTD. In this group, LWDs were reported by 63.6% of patients both pre- and post-injury, with average LWD of 3.9 pre-injury vs. 19.8 days post-injury (*p* < 0.05). STD was reported by 8.3% vs. 51.7% (*p* < 0.001) of patients in the 12 months pre- and post-burn injury, with average days of STD being 5.0 vs. 33.2 days (*p* < 0.05). LTD was reported by 0.0% vs. 6.7% (*p* < 0.05) of patients in the 12 months pre- and post-burn injury, with the average days of LTD being 0 vs. 1.6 days (Table 3). A breakdown of the study cohort by region is provided in Supplementary Table S1. No significant differences were observed for the workdays missed, LTD, and STD across the different regions of the United States, as shown in Supplementary Table S1. Workers’ compensation data are provided in Supplementary Table S2.

## 4. Discussion

Our study quantified the lost workdays and utilization of short-term disability (STD) and long-term disability (LTD) leave in a US working-age population. This work demonstrates that all three are higher after a burn injury compared to the baseline lost workdays, as well as STD and LTD utilization, in the same population in the year prior to burn injury. Additionally, as expected, the need for a skin grafting operation resulted in the increased utilization of STD and LTD.

Previous studies in burn patients have demonstrated that the return to work is influenced by multiple factors, including burn severity, total body surface area (TBSA) burned, number of operative procedures, substance abuse disorders, length of stay (LOS), and mental disorders; however, the direct impact on STD and LTD use has not been previously quantified [18,21,22,23]. Burn patients who were not engaged in employment reported a lower overall health-related quality of life (HRQoL) based on both the generic EuroQol Five-Dimension Scale (ED-5D) and the Burn-Specific Health Scale (BSHS) measures at all assessment points. Historically, it is crucial to identify the risk factors that create barriers to return to work, which is vital for the creation of personalized work-related rehabilitation focused on improving outcomes for burn survivors [23].

Both children and adult burn patients experience high levels of chronic psychological trauma after a burn injury [9,24]. In contrast to adults, pediatric burn patients typically experience a swift return to their daily activities. Studies have shown that children regularly return to school within weeks of their burn injury [25]. There are various factors that may contribute to this rapid reintegration, such as the physical resilience of children, strong family support systems, and school counseling. Additionally, young burn survivors often receive specific accommodations and resources when they return to school, which can shorten recovery time. Working-age adults, however, may struggle with more severe psychological barriers, physical impairments, and job security concerns, and they may benefit from targeted programs for work entry [8]. Altered self-image and heightened bodily awareness are commonly experienced by burn survivors, which can lead to low confidence in their ability to return to work [26]. Bodily disfigurement, especially involving the head and face, can lead to psychological distress, which further complicates the return to a professional role in the workplace [27].

After a burn injury, unique challenges regarding sex have also been observed. Research has shown that young, female burn survivors who have undergone hospitalization and skin grafting procedures are at a significantly higher risk of facing academic difficulties compared to their male counterparts. This disparity stems from multiple factors, such as the psychological impact of scars, societal expectations regarding appearance, and possible bias after returning to work [28]. These findings highlight the need for targeted interventions in young females following a burn injury.

In a cohort of 104 burn patients, 70% of previously employed patients returned to work within 3 months of their injury. This percentage increased to 92% within 14 months, but 8% had not returned by the 24-month follow-up period. Overall, 67% of burn patients had resumed their work 4.5 years post-burn [23]. This illustrates the extent of the impact of burns on rehabilitation and return-to-work times among burn survivors. The previous studies have shown that the duration of time taken off work varies greatly among burn survivors, with some returning within weeks while others require several months to recover. This literature emphasized that recovery trajectories have a high degree of heterogeneity [29]. In a Finnish cohort, 78% of burn patients returned to the workplace within the first year after the burn injury, which emphasizes the potential for a smooth return to work when institutional support is available [23]. Additionally, a positive correlation has been found between returning to work and higher HRQoL scores, highlighting the idea that employment can have a therapeutic effect on the recovery process [30]. Of course, the relationship observed could be bidirectional in that those who have higher QoL scores are more likely to return to work.

A difference in return-to-work rates has also been identified between patients who were burned at work and those burned in non-occupational settings. The patients who were burned at the workplace suffered an overall unemployment rate of 44% one-year post-injury, while those injured in non-occupational settings had a 22% unemployment rate one-year post-injury [31]. This difference in unemployment rates underscores the substantial impact of workplace injuries on long-term outcomes, as workplace injuries may be more severe than those sustained in non-occupational settings, and there may be an impact on the amount of rehabilitation required to return to the types of occupations in which a burn injury could be sustained, i.e., more physically strenuous or dexterous occupations.

Estimates of overall lost work productivity due to non-fatal injury in the US vary highly by injury mechanism and condition. Average lost work days due to injury were reported to be 11 days, ranging from 1.5 days for minor injuries such as bites and stings to 44.1 days for more major injuries such as motorcycle crashes [32]. However, these average lost workdays were attributed to injury by comparing to the controls based on age, comorbid conditions, and insurance type. In our study, we relied only on the burn injury patient cohort to assess the baseline lost workdays by examining the period 12 months prior to injury and hence the 3 incremental days of lost work (with 8 days lost on average in the 12 months post-burn injury compared to 5.2 days for all injury/illnesses in the 12 months pre-burn injury, *p* < 0.05) attributable to burn injury.

Substantial economic burden is seen in other unexpected injuries. For example, there is an estimated 1-year economic loss due to lower extremity fracture exceeding $64,000 per patient and over 1700 h of work lost [19]. Chronic conditions are also highly detrimental for the working age population, with adults with arthritis/diabetes/heart disease exceeding 14 days of lost work, diabetes/heart disease exceeding 12 days, and adults with arthritis/heat disease/hypertension exceeding 10 days of missed work on average [33]. In the MarketScan^®^ Health and Productivity Management Database patient cohort, burn injury leads to more STD utilization by affected adults than a diagnosis of pulmonary arterial hypertension but less utilization of STD than a cancer diagnosis within the first year [34,35].

Our study does have a few limitations, a number of which are inherent to utilizing claims databases for studying burn patients. The first is that a number of patients have missing data, and those cannot be imputed. For example, while we had 1079 patients with burn injuries in the worker’s compensation file, fewer than 10 had information provided for indemnity payments, limiting our ability to include these data. This bring us to our second, and notable limitation, which is that the database and hence the study only included data for insured individuals, which may limit the applicability of the findings to uninsured or under-insured populations, who tend to be more vulnerable with more limited access to healthcare. Data contained in the MarketScan^®^ databases comes primarily from large employers, also potentially underrepresenting medium and small companies and their employees. Most large databases have limitations that affect generalizability. While employing a national database in this study, such as The Healthcare Cost and Utilization Project (HCUP), would allow us to study additional populations at different income levels, it would not allow us to study short-term or long-term disability, time away from work, and other variables of interest in our study as those are not captured by other databases [36]. Furthermore, all regions of the country are not uniformly represented within this dataset [20]. Moreover, while ideally we would be able to quantify the amount of lost work and the utilization of STD and LTD by burn size and severity, the cohort had a significant number of individuals (approximately 90% of the cohort) with the total body surface area burned not captured by ICD-10 codes. Thus, we could not stratify the data by injury severity without losing power. This is unfortunately a function of what is coded in the medical record, shared with the insurers, and then added to the database. Lastly, while we have captured lost workdays, and long- and short-term disability leave, there is not a measure of reduced work hours or changes in work duties, which may be a direct result of the burn injury but is not captured in the database.

## 5. Conclusions

This study quantifies the increases in utilization of both short-term and long-term disability in the period following a burn injury for an adult, insured, working population, showing that the utilization of both increase post-burn injury. It also quantifies the economic implications of a burn injury, such as lost work in the 12 months following burns. Further study is needed to understand the total economic impact of burn injury on patients, businesses, and payor programs.

## Figures and Tables

**Table 1:** Demographics for Burn Cohort (2018–2019).

Age	
18–24	82 (4.7%)
25–44	910 (52.2%)
45–64	752 (43.1%)
**Sex**	
Female	667 (38.2%)
Male	1078 (61.8%)
**Region**	
South	718 (41.2%)
North Central	501 (28.7%)
West	284 (16.3%)
Northeast	240 (13.8%)
Unknown	2 (0.11%)
**Plan Type**	
Low Cost Sharing	241 (13.9%)
Average Cost Sharing	775 (44.8%)
High Cost Sharing	716 (41.3%)
**Employment Status**	
Full-time	1561 (89.5%)
Other	99 (5.7%)
Part time	70 (4.0%)
Cobra	15 (0.9%)
Retiree	0
**Discharge Disposition ^1^**	
Home	140 (78.2%)
Home Health	14 (7.8%)
Other	14 (7.8%)
Other Facility	7 (3.9%)
Rehab	3 (1.7%)
Skilled Nursing Facility	1 (0.56%)
Long-Term Acute Care Facility	0
Death/Hospice	0
**Comorbidities**	
0	1634 (93.6%)
1–2	90 (5.2%)
≥3	21 (1.2%)
**Year**	
2018	940 (53.9%)
2019	805 (46.1%)

^1^ For sub-cohort only.

**Table 2:** Lost workdays and short-term disability (STD) and long-term disability (LTD) utilization in privately insured patients with burn injury, 2018–2019.

	Sample Size (n)	12 m Pre (min, max)	12 m Post (min, max)	Delta	*p*-Value
Any Lost Work (%)	263	50.6%	53.6%	3.0%	0.2285
Days missed (days)	263	5.15 (0, 77)	7.99 (0, 243)	2.84	0.0145
Any STD (%)	1449	8.1%	20.3%	12.2%	<0.0001
Days STD (days)	1449	3.70 (0, 198)	9.34 (0, 301)	5.64	<0.0001
Any LTD (%)	1448	0.0%	1.0%	1.0%	0.0001
Days LTD (days)	1448	0 (0, 1)	2.01 (0, 426)	2.01	0.0019

**Table 3:** Lost workdays and short-term disability (STD) and long-term disability (LTD) utilization in privately insured patients with burn injury requiring skin grafting, 2018–2019.

	Sample Size (n)	12 m Pre (min, max)	12 m Post (min, max)	Delta	*p*-Value
Any Lost Work (%)	11	63.6%	63.6%	0.0%	1
Days missed (days)	11	3.9 (0, 14)	19.8 (0, 68)	15.9	0.0295
Any STD (%)	60	8.3%	51.7%	43.4%	<0.0001
Days STD (days)	60	5.0 (0, 102)	33.2 (0, 296)	28.2	0.0008
Any LTD (%)	60	0.0%	6.7%	6.7%	0.0445
Days LTD (days)	60	0 (0, 0)	1.6 (0, 44)	1.6	0.0905

## Data Availability

The study’s findings are based on data from the IBM^®^ MarketScan^®^ Commercial Claims and Health and Productivity Management Databases. Due to privacy and legal constraints, the data are not accessible to the general public. Nevertheless, the data can be provided upon a reasonable request from the corresponding author and with the necessary permissions from IBM Watson Health.

## Supplementary Materials

The following supporting information can be downloaded at: https://www.mdpi.com/article/10.3390/ebj5040041/s1, Figure S1: title; Table S1: Burn Cohort days missed, short-term disability (STD), and long-term disability (LTD) by region of the United States; Table S2: Burn Cohort Indemnity, Medical, Other, and Total Payments for those with payment information available.
