# Supplementary material for: Lost Work Due to Burn-Related Disability in a US Working Population
Source: Eur Burn J. 2024 Dec 19;5(4):464–73. doi: 10.3390/ebj5040041 (PMC11726730; doi:10.3390/ebj5040041)
Supplement: Supplementary file 1 [file ebj-05-00041-s001.zip › ebj-3241101-supplementary.pdf]

Supplementary Table 1: Burn Cohort days missed, short-term disability (STD), and long-term disability (LTD) by region of the United States

	Sample Size	Region				p-value
		Northeast	North Central	South	West	
**Days Missed (day)**	263	6<br>(0, 68)	10<br>(0, 243)	8<br>(0, 87)	6<br>(0, 26)	0.5862
**Days Missed (for those who missed days)**	141	11<br>(3, 68)	20<br>(1, 243)	14<br>(1, 87)	10<br>(1, 26)	0.3153
**Days STD (day)**	1447	7<br>(0, 247)	12<br>(0, 301)	9<br>(0, 296)	7<br>(0, 140)	0.0981
**Days STD (for those who took STD)**	294	40<br>(7, 247)	47<br>(2, 301)	44<br>(1, 296)	62<br>(8, 140)	0.2256
**Days LTD (day)**	1446	0.1<br>(0, 29)	2.8<br>(0, 302)	1.8<br>(0, 426)	2.8<br>(0, 426)	0.579
**Days LTD (for those who took LTD)**	15	29<br>(29, 29)	231<br>(126, 302)	209<br>(3, 426)	170<br>(1, 426)	0.6868

Supplementary Table 2: Burn Cohort Indemnity, Medical, Other, and Total Payments for those with payment information available

	Sample size (n)	12m pre	12m post	Delta	p-value	Median	IQR
**Indemnity Payment**	*	*	*	*	*	*	*
**Medical Payments**	19	.	$3,015	.	.	$460	$1,654
**Other Payments**	16	.	$632	.	.	$67	$610
**Total Payments**	21	.	$5,874	.	.	$486	$2,788
**Workdays Absent**	13	.	41	.	.	22	55

*** Findings were not included if sample size < 10**
